# Psychiatric discharge management practice in Europe – a systematic search and review

**DOI:** 10.1192/j.eurpsy.2025.1604

**Published:** 2025-08-26

**Authors:** S. Lech, D. Marbin, C. Hering, R. Kohl, J. Supplieth, J. O’Sullivan, S. Schreiter

**Affiliations:** 1 Department of Psychiatry and Neurosciences; 2 Institute of Medical Sociology and Rehabilitation Science, Charité - Universitätsmedizin Berlin, Berlin, Germany

## Abstract

**Introduction:**

Effective discharge management is essential for psychiatric inpatient care, significantly impacting patient outcomes during and after the transition from inpatient to outpatient settings. A well-coordinated discharge process ensures continuity of care, reduces readmission risks, and supports long-term recovery. However, there is a notable lack of comprehensive data on discharge practices across European countries, impeding the evaluation of their effectiveness and the development of informed improvements.

**Objectives:**

The present study has two primary objectives: first, to systematically review current practices, challenges, and outcomes related to psychiatric discharge management across European countries. Second, to evaluate and compare existing discharge guidelines, protocols, and toolkits. By synthesizing these findings, we aim to identify best practices, highlight gaps, and offer recommendations for optimizing discharge procedures. Additionally, we will briefly present a current mixed-method research project from Germany, titled “*Evaluation of Discharge Management in Psychiatric Care*,” which aims to assess discharge management practices for psychiatric inpatients. These insights will supplement the broader European review by offering a focused perspective on the practical considerations and challenges of discharge management practices within a specific national context.

**Methods:**

We will conduct a systematic search and review by exploring multiple electronic databases (MEDLINE, Embase, Cochrane Library and PsycINFO) and grey literature sources (Google and Google Scholar) for both quantitative (observational studies, reports) and qualitative (clinical guidelines, protocols, interviews, focus groups) data related to psychiatric discharge management. Additionally, we will hand-search the references of key papers, including existing systematic reviews and included articles. The search will include documents published from 2000 to December 31, 2024.

**Results:**

The presentation will provide an overview of the current research and literature on psychiatric discharge management practices in Europe. The review is expected to provide critical insights into advancing psychiatric care standards. Additionally, the presentation aims to engage researchers and clinical practitioners attending EPA25 by providing a platform for knowledge exchange on psychiatric discharge management, facilitating networking opportunities and fostering potential collaborations for future research in this vital field.

**Conclusions:**

Discharge management is a critical aspect of psychiatric care that requires meticulous planning and coordination. The insights gained from this review will contribute to the development of evidence-based discharge protocols that are adaptable to diverse patient populations and healthcare settings, ultimately leading to more effective and cohesive care strategies across Europe.

**Disclosure of Interest:**

None Declared

